# Genetic Insights into Primary Restrictive Cardiomyopathy

**DOI:** 10.3390/jcm11082094

**Published:** 2022-04-08

**Authors:** Andreas Brodehl, Brenda Gerull

**Affiliations:** 1 Erich and Hanna Klessmann Institute, Heart and Diabetes Center NRW, University Hospital of the Ruhr-University Bochum, Georgstrasse 11, 32545 Bad Oeynhausen, Germany; 2 Comprehensive Heart Failure Center (CHFC), Department of Medicine I, University Clinic Würzburg, Am Schwarzenberg 15, 97078 Würzburg, Germany

**Keywords:** restrictive cardiomyopathy, cardiomyopathy, cardiovascular genetics, desmin, troponin, filamin-C

## Abstract

Restrictive cardiomyopathy is a rare cardiac disease causing severe diastolic dysfunction, ventricular stiffness and dilated atria. In consequence, it induces heart failure often with preserved ejection fraction and is associated with a high mortality. Since it is a poor clinical prognosis, patients with restrictive cardiomyopathy frequently require heart transplantation. Genetic as well as non-genetic factors contribute to restrictive cardiomyopathy and a significant portion of cases are of unknown etiology. However, the genetic forms of restrictive cardiomyopathy and the involved molecular pathomechanisms are only partially understood. In this review, we summarize the current knowledge about primary genetic restrictive cardiomyopathy and describe its genetic landscape, which might be of interest for geneticists as well as for cardiologists.

## 1. Introduction

In clinical practice, cardiomyopathies are divided according to structural and functional criteria into different classes [1,2]. Classification according to their etiology revealed a non-negligible percentage of genetic cases for all structural cardiomyopathies [3]. In comparison to hypertrophic cardiomyopathy (HCM) with an estimated prevalence of 1:500 [4], the prevalence of restrictive cardiomyopathy (RCM) is currently unknown [5]. However, because of the rarity of primary RCM, its genetic background is poorly defined compared with other cardiomyopathies. Beside primary RCM, it can manifest as a part of systemic diseases such as amyloidosis [6], which can also be genetically caused, for example, by mutations in the *TTR* (transthyretin) gene [7]. In addition, RCM can also be part of different syndromic diseases, e.g., Alström syndrome (MIM, #203800) [8] or Myhre syndrome (MIM, #139210) [9]. In this review, we will focus on the genetic etiology of primary RCM and will summarize the current knowledge of the RCM-associated genes.

## 2. Clinical Description

**Figure 1:**
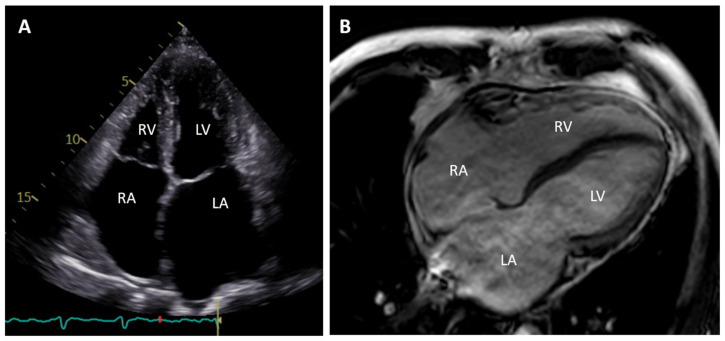
(**A**) Apical four chamber view during systole of an echocardiogram (**B**) and four chamber view of cardiac magnetic resonance image of a 50-year-old patient carrying a pathogenic *FLNC* mutation. Note the enlarged atria, normal ventricular sizes and wall thicknesses. RA = right atrium; RV = right ventricle; LA = left atrium; LV = left ventricle.

RCM is characterized by severely enlarged atria, normal-sized ventricles, with increased myocardial stiffness leading to impaired ventricular filling and diastolic dysfunction (Figure 1). Systolic function and ventricular wall thicknesses are often normal. Patients present with symptoms of left and/or right ventricular heart failure with preserved ejection fraction (HFpEF), atrial fibrillation, ventricular arrhythmias and frequently conduction disorders [10]. The overall prognosis is poor and the 5-year survival rate of adult patients with a confirmed genetic cause was 56% [11]. Specific therapies of non-infiltrative genetic forms do not exist. Non-specific therapies include fluid and sodium restrictions and medical treatment of heart failure with reduction of volume overload as well as anticoagulation and antiarrhythmic therapy. Very often heart transplantation (HTx) is the only option for long-term survival [12].

## 3. Genetic Landscape of Restrictive Cardiomyopathy

**Figure 2:**
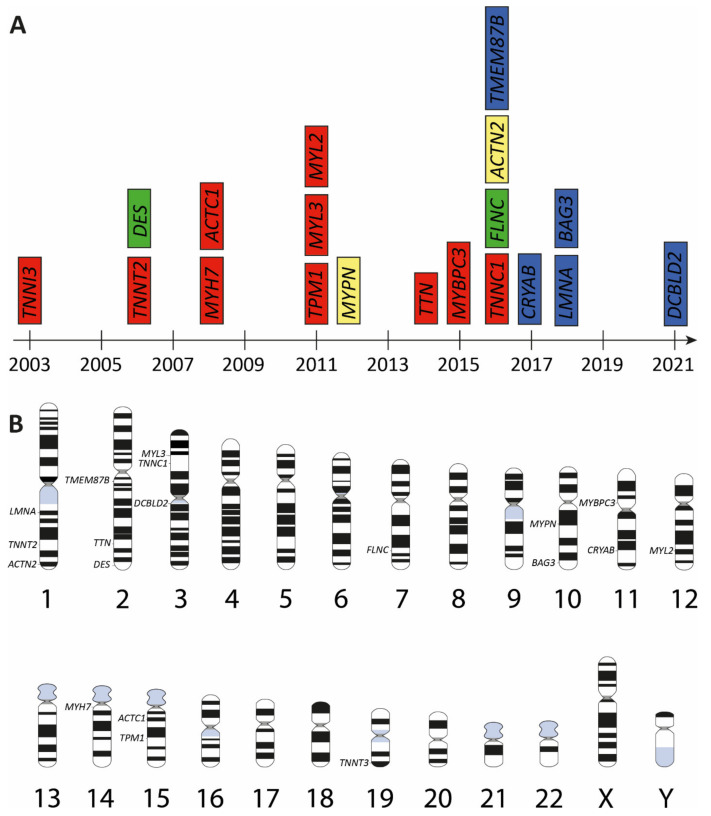
Overview of RCM genes. (**A**) Genes associated with restrictive cardiomyopathy (RCM) according to the year of discovery. Different subcellular localizations are color-coded (red = sarcomere; green = cytoskeleton; yellow = Z-disc and blue = others). (**B**) Chromosomal location of RCM-associated genes. Schematic idiograms were licensed from shutterstock.de.

**Figure 3:**
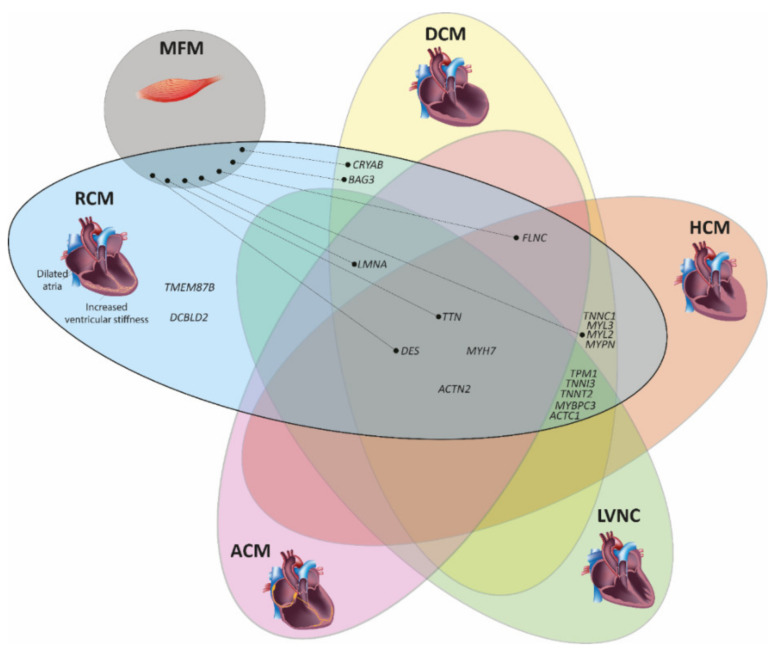
Venn diagram showing the genetic overlap of restrictive cardiomyopathy (RCM) with other cardiomyopathies. ACM = arrhythmogenic cardiomyopathy; DCM = dilated cardiomyopathy; HCM = hypertrophic cardiomyopathy; LVNC = left ventricular non-compaction cardiomyopathy; and MFM = myofibrillar myopathy. Gene names according to the HUGO Gene Nomenclature Committee, HGNC (https://www.genenames.org/ (accessed on 13 March 2022)). Sub-images of the DCM or HCM heart were licensed from shutterstock.de.

Currently, pathogenic mutations in 19 different genes have been identified in patients with RCM (Table 1 and Figure 2A). Since RCM is a rare cardiomyopathy with an unknown prevalence [13], the genetic landscape is not completely discovered. At present, for several of the known RCM genes, only a single family or even a single index patient has been reported. All known RCM genes are localized on autosomes (Figure 2B) and in most cases, the mutations are inherited in an autosomal dominant mode or appear as de novo mutations. However, there are also some examples for a recessive inheritance pattern [14]. The majority of RCM genes encode for sarcomere, cytoskeleton or Z-disc proteins, e.g., the cardiac troponins, desmin or filamin-C (Figure 2A). Remarkably, there is a significant genetic overlap with other cardiomyopathies especially with HCM and to some extent with dilated cardiomyopathy (DCM), left-ventricular non-compaction cardiomyopathy (LVNC) or arrhythmogenic cardiomyopathy (ACM) (Figure 3). Currently, it is unknown why mutations in the same gene cause different cardiomyopathies. However, additional genetic modifiers as well as diverse environmental factors can be suggested to be contributing to these phenotypical differences. Sometimes, different phenotypes including RCM are even present within the same family [15,16].

Remarkably, there is also a genetic overlap between RCM and myofibrillar myopathy (MFM, MIM, #601419). MFM is a group of genetic muscle diseases characterized by myofibrillar disorganization and abnormal intra-sarcoplasmic protein aggregates [17]. It can affect the skeletal and/or cardiac muscle. Mutations in seven genes cause MFM (*DES* [18], *CRYAB* [19], *FLNC* [20], *LMNA* [21], *BAG3* [22], *TTN* [23,24], *MYL2* [25]) as well as RCM (Table 1). The genetic overlap between both diseases (Figure 3) might indicate a detrimental involvement of pathological cardiac protein aggregates [26].

**Table 1:** Overview about RCM-associated genes and proteins.

Gene	Cytogenetic Location	Encoded Protein	Subcellular Protein Localization	First Description	References
*TNNI3*	19q13.42	cardiac troponin I	Sarcomere	2003	[27]
*TNNT2*	1q32.1	cardiac troponin T	Sarcomere	2006	[28]
*DES*	2q35	desmin	Intermediate filament	2006	[29]
*ACTC1*	15q14	cardiac actin	Sarcomere	2008	[30]
*MYH7*	14q11.2	β myosin heavy chain	Sarcomere	2008	[31]
*TPM1*	15q22.2	tropomyosin 1	Sarcomere	2011	[32]
*MYL3*	3p21.31	essential myosin light chain 3	Sarcomere	2011	[32]
*MYL2*	12q24.11	cardiac regulatory myosin light chain	Sarcomere	2011	[32]
*MYPN*	10q21.3	myopalladin	Sarcomere, Z-disc	2012	[33]
*TTN*	2q31.2	titin	Sarcomere	2014	[34]
*MYBPC3*	11p11.2	cardiac myosin binding protein C	Sarcomere	2015	[35]
*TNNC1*	3p21.1	cardiac troponin C	Sarcomere	2016	[36]
*FLNC*	7q32.1	filamin C	Intercalated disc, Z-disc, sarcolemma	2016	[37]
*TMEM87B*	2q13	transmembrane protein 87 B	Membrane	2016	[38]
*ACTN2*	1q43	α actinin 2	Z-disc	2016	[39]
*CRYAB*	11q23.1	αB crystallin	IF associated protein, intercalated disc, Z-disc	2017	[40] ^1^
*LMNA*	1q22	lamin A/C	Nuclear lamina	2018	[41]
*BAG3*	10q26.11	bcl2 associated athanogene 3	Cytosol	2018	[42]
*DCBLD2*	3q12.1	discoidin cub and lccl domain containing protein 2	Membrane	2021	[43] ^2^

^1^ RCM-associated with skeletal myopathy. ^2^ RCM-associated with atrial fibrillation, tachycardia, developmental delay and dysmorphic features.

### 3.1. Mutations in Genes Encoding for Sarcomere Proteins

The majority of known RCM-associated mutations are found in ten genes encoding for sarcomere proteins (Figure 2A). These mutations affect the thin and thick filaments as well as titin filaments.

#### 3.1.1. Cardiac Troponins (TNNI3, TNNT2, TNNC1) and Alpha-Tropomyosin (TPM1)

The cardiac troponin complex is composed of three subunits controlling the position of tropomyosin, essential for the regulation of striated muscle contraction and located along the sarcomere thin filament [44]. Disruption of regulatory function due to mutations leads to cardiac dysfunction and cardiomyopathy. Since the early 1990s, cardiac troponins are known as disease genes for HCM [45], however, they expand their disease spectrum to all genetic forms of cardiomyopathies including RCM.

**Figure 4:**
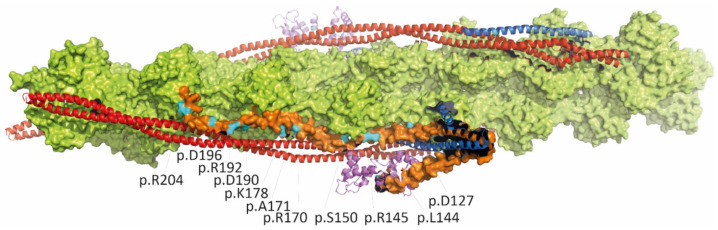
Schematic molecular structure of the thin filaments in the Ca^2+^ free state [68] (https://www.rcsb.org/structure/6KN7 (accessed on 13 March 2022)). Actin is shown in light green, tropomyosin is shown in red, cardiac troponin T is shown in blue, troponin C is shown in violet and troponin I is shown in orange. The localizations of the RCM-associated *TNNI3* missense mutations are shown in cyan. The majority of RCM-associated *TNNI3* missense mutations are localized in the C-terminal part of troponin-I.

The gene encoding the cardiac isoform of troponin I (TNNI3) is the main target gene for RCM within the thin filaments and the sarcomeres. Almost all mutations are located in the regulatory C-terminal region interacting with actin and the N-terminal domain of TNNC1 (Table 2 and Figure 4). A high proportion of de novo mutations in infants and children with a poor outcome are described. Few mutations are solely reported to cause an RCM phenotype, but most of them are also found in patients with HCM. Studies on skinned fibers by Gomes et al. suggest that TNNI3 mutations increase Ca^2+^ sensitivity of force development and decrease the ability of TNNI3 to inhibit actomyosin ATPase activity, leading to impaired relaxation properties and diastolic dysfunction [46]. Additionally, it has been shown that mutant alleles, such as p.L144Q, p.R145W and p.R170W, incorporate into the thin filaments to a lower extent compared to wildtype affecting the structural stability of the filaments [47,48]. Overall, it appears that similar mutations can cause a hypertrophic, dilated or restrictive phenotype assuming that genetic modifiers or other environmental factors influence the age of onset and phenotypic expression. A transgenic mouse model (cTNI-193His) corresponding to the human p.R192H mutation mimics the RCM phenotype in mice and suggests that impaired relaxation resulting from Ca^2+^ hypersensitivity [49] and diastolic dysfunction occurring in a dose-dependent manner and indicating that the dosage of mutant protein may be important for the severity of impaired diastole [50].

In contrast to TNNI3, a restrictive phenotype appears to be less common in the two other troponin genes. Mutations in TNNT2 are mainly reported in rare cases where other cardiomyopathy phenotypes also occur in the same family. Furthermore, two compound heterozygous mutations in the cardiac TNNC1 evolved in a restrictive phenotype in two infants (Table 2) [36]. Kawai et al. developed a knock-in mouse model (TnC-A8V), which mimics the human phenotype of enlarged atria, hyper contractility and diastolic dysfunction. The authors suggest perturbed cross-bridge kinetics by myosin rod hypophosphorylation as a potential novel mechanism [51].

Alpha tropomyosin (encoded by TPM1) is a long, double-stranded, helical coiled-coil protein that is wrapped about the long axis of the actin backbone (Figure 4, red structure) and serves to block the active site on actin, thereby inhibiting actin and myosin from binding under resting conditions. TPM1 and the troponin complex constitute the Ca^2+^-sensitive switch that regulates the contraction of cardiac muscle fibers. Several missense mutations have been described causing either HCM or DCM [52]. Recently, Dorsch et al. reported a 6-year-old child with severe RCM carrying two TPM1 variants in compound heterozygous state requiring HTx, whereas family members with one of the two variants expressed an HCM-like phenotype [16]. In summary, the one case indicates that TPM1 is a very rare disease gene and the RCM phenotype may only occur in compound heterozygosity.

**Table 2:** Overview about known RCM-associated thin filament mutations.

Mutation	Age of Onset and Clinical Features	Family History	MAF ^1^	Comments	References
*TNNI3*					
p.D127Y	infant, HF, VAD	de novo	-	contractile dysfunctions and effects on thin filament structure	[53]
p.L144Q	adult, HF	unknown	-		[27]
p.L144H	young adults, HF	familial	-		[54]
p.R145W	children and adults, HF	familial, autosomal dominant	3/280226	variant also associated with HCM; Dutch founder mutation; segregation in several families	[27,39,55]
p.R145Q	children	familial, far relative HCM	-	associated with HCM	[55]
p.S150P	child, SCD	familial	-	one Chinese family with several affected members	[56]
c.549+2delT	infant, died at age 2	de novo	-	predicts splicing defect and truncation	[55]
p.D168fsX176	child, HF, died at age 28y	de novo	-	protein reduction	[57]
p.R170G	child, HF	de novo	-		[47]
p.R170W	infant	de novo	-	variant also associated with HCM	[47,58]
p.R170Q	child, HF	de novo	-	variant also associated with HCM	[30,54]
p.A171T	adult, HF, AF	unknown	-		[27]
p.E177fsX209	child	de novo	-		[30]
p.K178E	6y, HF	de novo	-		[27]
p.K178del	child	de novo	-		[55]
p.D190H	mainly adults, HF, SCD	familial	-	named in ClinVar as p.D190G	[27]
p.R192C	child	familial	-	carries also mosaicism of p.R145Q; associated also with HCM in far relative	[55]
p.R192H	children, young adult, HF	de novo	-	independent reports of de novo mutations; variants also associated with HCM	[27,59,60]
p.K193E	adults, AF, SCD	familial	-	cousin developed HCM	[61]
p.I195fs	young adult, HF, HTx	de novo	-	dominant-negative effect	[62]
p.D196H	three adults, HF, HTx	familial, homozygous	-	heterozygous carrier asymptomatic	[63]
p.R204H	children, HF, HTx, VSD in one case	de novo	-	independent reports of de novo mutations	[59,64,65]
*TNNT2*					
p.I89N	two adult cases within one family	familial	0.00002	mixed phenotype with HCM and DCM	[66]
p.R104C	children, young adult, HF	familial	-	mixed phenotype with HCM in the family	[67]
p.E69del	infant, HF, VAD	de novo	-		[28]
p.E146K	child	familial	0.00003	variant also associated with other CMPs	[30]
*TNNC1*					
p.A8V; p.D145E	two infants died	familial, compound heterozygous	0.00001 0.0001	HCM which evolved into RCM	[36]
*TPM1*					
p.E62Q; p.M281T	child	familial, compound heterozygous	- 0.00001	each single variant leads to a HCM like phenotype	[16]
*ACTC1*					
p.D313H	child	familial	-	father was diagnosed with DCM	[30]

^1^ MAF = Minor allele frequency according to Genome Aggregation Database (February 2022), https://gnomad.broadinstitute.org (accessed on 13 March 2022). AF = atrial fibrillation, CMPs = cardiomyopathies, DCM = dilated cardiomyopathy, HCM = hypertrophic cardiomyopathy, HF = heart failure, HTx = heart transplantation, RCM = restrictive cardiomyopathy, SCD = sudden cardiac death, VAD = ventricular assist device, VSD = ventricular septal defect.

#### 3.1.2. Cardiac Actin (ACTC1)

Human cardiac α-actin, encoded by ACTC1, is one of the six human actin isoforms. Using fluorescence in situ hybridization technique Ueyama et al. showed that ACTC1 is localized on chromosome 15q14 [69]. Cardiac α-actin is highly conserved between different species and skeletal and cardiac α-actin are co-expressed in cardiomyocytes [70]. As a monomer, actin has a globular structure (G-actin) and polymerize into filaments (F-actin). Actin is the major structural component of the thin filaments (Figure 4, green structure) and is eminent for the contraction cycle and force generation of cardiomyocytes [71].

Kaski et al. described for the first time an RCM causing mutation in ACTC1 (p.D313H) [30]. The father developed DCM and the sister of the index patient showed a mixed RCM/DCM phenotype, but no genetic sequence analysis was performed for both [30]. Functional analysis was not performed in this study. However, ACTC1-p.D313H is localized in the tropomyosin binding region which supports its functional impact. In addition, ACTC1 mutations can cause DCM [72], HCM [73], LVNC [74] and septal defects [75] (Figure 3).

#### 3.1.3. Myosin Heavy and Light Chains (MYH7, MYL2 and MYL3)

The thick filaments of the cardiac and skeletal sarcomere are mainly formed by myosin. Human cardiac myosin is a hexameric protein complex consisting of β myosin heavy chains (encoded my MYH7), two essential light chains (encoded by MYL3) and two regular myosin light chains (encoded by MYL2) [76,77,78]. Myosin proteins consist of a head, neck and tail domain. The head domains interact with the thin filaments and contain the N-terminal globular motor domains [79] performing the power stroke during contraction [80]. The neck region is bound by the myosin light chains [81] and the tail domains build a coiled-coil [82].

In all three myosin genes, mutations have been found in RCM patients (Table 3). For the first time, Karam et al. described in 2008 a de novo mutation in the MYH7 gene (p.P838L) in an infantile patient with RCM [31]. Several further pathogenic MYH7 mutations have been described for RCM (Table 3). The majority of these mutations are missense mutations. Beside RCM, MYH7 mutations are particularly causative for HCM [83] and to a less extent for DCM [84], LVNC [74] and ACM [85].

In 2011, Caleshu et al. reported a female RCM patient carrying MYL2-p.G57E and in addition MYL3-p.E143K^hom^ [32]. The described index patient carrying these myosin light chain variants do not present a family history of cardiomyopathies [32], which might be caused by a recessive inheritance. The mutation MYL3-p.E143K^hom^ was also identified before in the homozygous state in HCM patients [86]. Transgenic mice with the cardiac expression of human MYL3-p.E143K developed an increased ventricular stiffness, cardiac interstitial fibrosis and showed ultrastructural defects of the sarcomeres leading to a restrictive phenotype [87]. *MYL2* and *MYL3* mutations also cause HCM [88,89] and DCM [90] (Figure 3).

**Table 3:** Overview about known RCM-associated myosin mutations (*MYH7, MYL2, MYL3*).

Mutation	Age of Onset and Clinical Features	Family History	MAF ^1^	Comments	References
*MYH7*					
p.Y386C	infant, coronary artery bridging	unknown	-		[91]
p.R721K	adult, AF,	familial	-	in combination with *ABCC9*-p.R1186Q	[92]
p.G768R	adult, AF,	unknown	-		[39]
	death at age 42				
	infant, HTx	unknown	-		[93]
p.R783H	adult, AVB,	familial	0.00002	son has HCM	[39]
	death at age 54				
p.P838L	infant	de novo	-		[31]
p.L840M	child	unknown	-	in combination with *MYBPC3*-p.P147L	[39]
p.R870C	two adults, AF	familial	0.00002	myofibrillar disarray,	[94]
				cardiomyocyte necrosis,	
				abnormal nuclei morphology	
p.I909M	adult, AVB, AF, death at age 56	unknown	-		[39]
p.T1188CfsX22	adult, in combination with LVH	de novo	-		[95]
*MYL2*					
p.G57E	adult	absent	0.000004	in combination with *MYL3*-p.E143K^hom^	[32]
*MYL3*					
*MYL3*-p.E143K^hom^	adult	absent	0.00001	in combination with *MYL2*-p.G57E	[32]

^1^ MAF = Minor allele frequency according to Genome Aggregation Database (February 2022), https://gnomad.broadinstitute.org (accessed on 13 March 2022). AF = atrial fibrillation, AVB = atrioventricular block, HCM = hypertrophic cardiomyopathy, HTx = heart transplantation, LVH = left ventricular hypertrophy, VUS = variant of unknown significance.

#### 3.1.4. Cardiac Myosin Binding Protein C (MYBPC3)

Another main disease gene for HCM and to a minor extent DCM and LVNC is the gene encoding the cardiac myosin binding protein C (MYBPC3). One study by Wu et al. showed that one de novo variant, previously also associated with HCM (p.E334K) and one truncation variant p.Q463X might cause RCM as part of the phenotypic spectrum [35].

#### 3.1.5. Titin (TTN)

Titin is the largest known human protein and represents the third filament system in cardiac and skeletal muscle [96]. Its primary role is maintaining sarcomere organization, generation of passive tension during muscle stretching and modulating contraction. The major cardiac phenotype caused by TTN mutations is DCM, however so far almost exclusively truncation variants are proven to be causative accounting for 30% of affected individuals with DCM [97,98]. Recently, multiple pathogenic mechanisms have been suggested including haploinsufficiency, truncated titin polypeptides as well as post-translational modifications of titin [99,100]. The role of missense variants is poorly understood, but at least for DCM their relevance as causative remains questionable; they may have a modifying effect [101]. Rarely, other cardiac phenotypes such as HCM, RCM and ACM have been suggested to be associated with TTN variants. In particular a de novo missense mutation, p.Y7621C, located in the A/I junction of titin has been shown to segregate in a family with five affected members aged 12–35 years with typical features of a restrictive physiology suggesting that other missense mutations may also relevant for RCM in particular if they appear de novo [34].

### 3.2. Mutations in Genes Encoding Non-Saromere Proteins

Although the majority of RCM-associated mutations has been found in genes encoding for different sarcomere proteins (Figure 2A), mutations in non-sarcomeric genes are also relevant. Several different mutations have been reported, for example in the DES and FLNC genes.

#### 3.2.1. Desmin (DES)

**Figure 5:**
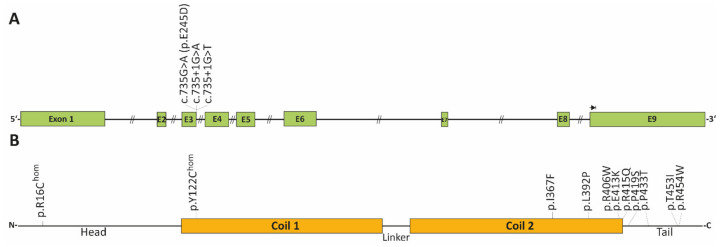
Schematic overview of RCM associated DES mutations. (**A**) Schematic overview about the DES gene consisting of nine exons (NM_001927.4). Three splice site mutations have been identified in RCM patients at the donor splice site of exon 3. (**B**) Schematic domain organization of desmin and the localization of the known RCM-associated DES missense mutations.

The DES gene encodes the cytoplasmic muscle specific intermediate filament protein desmin. Intermediate filaments connect different cell organelles such as the cardiac desmosomes, costameres, Z-discs, mitochondria and the cell nuclei [102,103]. Cardiac desmosomes are cell–cell junctions localized at the intercalated disc mediating the cell–cell adhesion of the cardiomyocytes [104]. Desmin filaments are coupled to the desmosomes via the cytolinker protein, desmoplakin [105]. Costameres are multi-protein complexes localized at the sarcolemma and connect the extracellular matrix with the myofibrils [106]. The intermediate filaments are connected via different cytolinker proteins, e.g., plectin with the Z-bands and the costameres [107]. Due to its central role in the cardiac intermediate filament system and its connections with several multi-protein complexes or cell organelles, desmin is highly relevant for the structural integrity of the cardiomyocytes. DES-deficient mice developed severe cardiomyopathy in combination with skeletal myopathy characterized by fragile myofibrils, severe cardiac fibrosis, cardiomyocyte necrosis and abnormal calcium deposits [108,109]. DES mutations in humans are associated with different skeletal and cardiac myopathies [110,111,112,113,114]. In 2006, Hager and colleagues described for the first time a patient with RCM carrying the mutation DES-p.E245D. Later, it was recognized that this mutation causes a splicing defect leading to an in-frame skipping of exon-3 causing a deletion of 32 amino acids within the rod domain [115,116]. Several other pathogenic RCM-associated DES mutations have been reported [14,117,118,119,120,121,122] (Figure 5 and Table 4).

Most of the DES mutations are missense or small in-frame deletion mutations leading to a detrimental effect on the filament assembly process [123,124]. The desmin monomer consists of a central α-helical rod domain flanked by non-helical head and tail domains [125]. Two desmin monomers form coiled–coil dimers driven by the annealing of a hydrophobic seam [126]. These dimers form anti parallel tetramers [127]. Eight tetramers anneal into unit-length filaments (ULFs) which have a size of about 60 nm [128]. ULFs are the essential building blocks of intermediate filaments and hybridize longitudinally into regular intermediate filaments [125,129]. As intermediate filaments do not have a polar orientation, they can fuse end-to-end [130,131,132]. DES mutations can disturb the filament assembly at different steps [123,124].

**Table 4:** Overview about known RCM-associated *DES* mutations.

Mutation	Age of Onset and Clinical Features	Family History	MAF ^1^	Comments	References
c.735+1G>A	adult, SM	de novo	-	induces a splice defect, skipping of exon-3	[133]
c.735+1G>T	adults, SM	two patients	-	induces a splice defect, skipping of exon-3	[119]
p.R16C	adult, AVB, HTx	one patient	0.000006570	homozygous	[134]
p.Y122H	adult, AVB	one patient	-	homozygous	[14]
c.735G>C (p.E245D)	adults, AF	several family members, only index patient was genotyped	-	induces a splice defect, skipping of exon-3	[116]
p.I367F	adults, AVB, SM	several family members	-	index patient diagnosed with HCM [135]	[15,135]
p.L392P	adult, AVB, SM	one patient	-		[135]
p.R406W	adults, AVB	three affected members	-	a different index patient presented ACM in combination with SM [112]	[117,134]
p.E413K	adults, AVB, AF, SCD	four affected members	-		[136,137]
p.R415Q	adult, AF	several family members	-	different phenotypes, unclear if a splice defect is caused (last bp of exon-6)	[15]
p.P419S	adults, AVB, SM	two patients	-		[135]
p.P433T	adult, AVB, SM	one patient	-		[120]
p.T453I	adult, AVB	de novo	-		[134]
p.R454W	adults, AVB, SM	two patients	-		[112]

^1^ MAF = Minor allele frequency according to Genome Aggregation Database, https://gnomad.broadinstitute.org/ (accessed on 13 March 2022). ACM = arrhythmogenic cardiomyopathy, AF = atrial fibrillation, AVB = atrioventricular block, HCM = hypertrophic cardiomyopathy, HTx = heart transplantation, SCD = sudden cardiac death, SM = skeletal myopathy.

#### 3.2.2. Myopalladin (MYPN)

Myopalladin belongs beside myotillin (MYOT) and palladin (PALLD) to the actin-binding and immunoglobulin-containing proteins within the Z-disc [138,139]. It contains five immunoglobulin (Ig) domains and a proline-rich motif [138]. In 2012, Purevjav et al. described a MYPN nonsense mutation (p.Q529X) in two affected siblings with RCM [33]. Beside RCM, MYPN mutations are also found in patients with DCM [140], HCM [141] and nemaline myopathy (MIM, #617336) [142].

#### 3.2.3. α-Actinin-2 (ACTN2)

**Figure 6:**
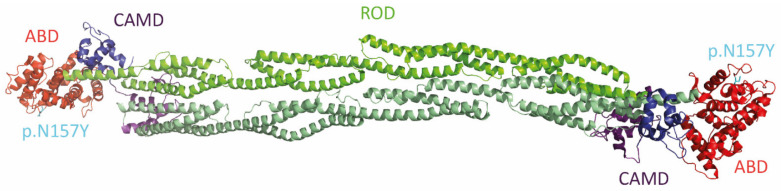
Structural overview of the anti parallel α-actinin-2 dimer (https://www.rcsb.org/structure/4D1E) (accessed on 13 March 2022) [149]. The N-terminal. Actin-binding domains are shown in red. Four spectrin-like repeats build the central cylindrical rod domain (green). A C-terminal calmodulin-like domain is built by two EF hand motifs (purple and blue). The position of the RCM-associated mutation ACTN2-p.N157Y within the actin-binding domain is shown in cyan.

The ACTN2 gene was mapped to chromosome 1q43 [143] and consists of 21 exons [144]. α-Actinin-2 is the main structural component of the Z-discs in striated muscles [145] and belongs to the spectrin protein family [146]. The typical structural element of this protein family are the spectrin-like repeats [147], which are formed by three α-helices forming a left-handed supercoil [148]. α-Actinin-2 forms anti parallel dimers and consists of an N-terminal actin binding domain, a central ROD domain and a calmodulin-like domain (CAMD) (Figure 6) [149]. 

In 2016, Kostareva et al. screened a cohort of 24 unrelated RCM patients using a broad cardiomyopathy next generation sequencing (NGS) panel and identified, among others, the likely pathogenic mutation ACTN2-p.N175Y (Table 1) [39]. Besides RCM, pathogenic mutations in ACTN2 are associated with DCM [150], HCM [151], LVNC [152] or ACM [153] indicating a broad spectrum of cardiac phenotypes associated with those mutations (Figure 3). In addition, ACTN2 mutations can also cause skeletal myopathies [154].

#### 3.2.4. Filamin-C (FLNC)

**Figure 7:**
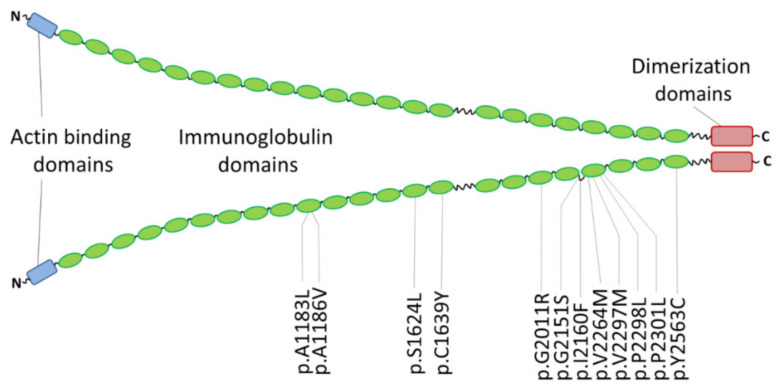
Schematic overview about the domain organization of filamin-C and the localization of the known RCM-associated FLNC missense mutations.

Originally, mutations in FLNC were identified in patients with MFM (MIM, #609524) [155] or distal myopathy (MIM, #614065) [156]. The FLNC gene consists of 48 exons and is mapped on human chromosome 7q32 [157]. It encodes filamin-C, which is a cytolinker protein. Filamin-C contains an N-terminal actin-binding domain and 24 immunoglobulin-(Ig) domains, which are separated by two hinge regions (Figure 7) [158]. The dimerization of filamin-C is mediated by a protein–protein interaction of its 24th Ig-domains [159]. In cardiomyocytes, filamin-C is localized at the intercalated discs, the sarcolemma and the Z-discs [158,160]. Several binding partners including titin [161,162], integrin β1A and myotilin [163] as well as actin and sarcoglycans [164] have been reported. For a detailed overview see [158].

Valdés-Mas et al. identified in 2014, by whole-exome sequencing several FLNC mutations in patients with HCM [165]. Of note, FLNC mutations can likewise cause DCM [166], ACM [167] or non-compaction cardiomyopathy [168]. RCM-associated FLNC mutations were described in two families for the first time in 2016 [37]. Since then, several other FLNC missense mutations have been identified in RCM patients (Table 5 and Figure 7). Whereas DCM-associated FLNC truncation mutations are presumably leading to haploinsufficiency [169], an abnormal aggregation leading in consequence to sarcomeric disarray has been demonstrated for some missense mutations [37,165,170]. Several (zebra)fish and mouse models for FLNC have been generated revealing different muscle and heart defects [166,171,172,173,174,175,176,177,178]. Of note, even in Drosophila melanogaster loss of the filamin ortholog ‘Cheerio’ causes Z-disc and sarcomere defects [162]. Recently, two iPSC lines from donors with RCM carrying FLNC missense mutations have been generated, but their characterization is ongoing [179,180]. Tucker et al. inserted the mutation FLNC-p.V2297M using genome editing by ‘Clustered Regularly Interspaced Short Palindromic Repeats’ (CRISPR)-Cas9 into a human embryonic stem cell line (hESC). The fractional shortening was decreased in hESC-derived cardiomyocytes [181].

#### 3.2.5. Lamin A/C (LMNA)

Lamin A/C belongs to the intermediate filament protein family (type V) [125] and forms the nuclear lamina [185]. The nuclear lamina is a molecular meshwork, which is important for the structural integrity of the nuclei and regulates the chromatin organization [186].

Recently, Paller et al. found a 1 bp deletion in exon 5 of the LMNA gene (c.835delG, p.E279RfsX201) in a RCM patient who developed additionally skeletal muscle weakness and atrial fibrillation [41]. Histology analysis revealed hypertrophy and cardiac fibrosis in the explanted myocardial tissue [41]. Beside RCM, LMNA mutations cause DCM [187], ACM [188], LVNC [189], Emery–Dreifuss muscular dystrophy (MIM, #181350) [190], familial lipodystrophy (MIM #151660) [191] and Hutchinson–Gilford progeria syndrome (HGPS, MIM #176670) [192]. The nuclear envelope and the connected nuclear lamina of cardiomyocytes are sensitive structures where mutations affect several other proteins, e.g., TMEM43 may cause different cardiomyopathies [193]. 

#### 3.2.6. Transmembrane Protein 87B (TMEM87B) 

TMEM87B encodes a multi-pass transmembrane protein, which is involved in endosome to Golgi apparatus retrograde transport [194]. 

Yu et al. described the hemizygous missense mutation TMEM87B-p.N456D in combination with a 1.7 Mb microdeletion on the second allele in a patient who developed RCM in combination with an atrial septal defect, craniofacial abnormalities, dysmorphic features, microcephaly and skeletal dysplasia [38]. Using anti sense morpholino injections, it has been shown by Russel et al. that TMEM87B knockdown causes cardiac hypoplasia and cardiac defects in zebrafish embryos [195]. 

#### 3.2.7. αB-Crystallin (CRYAB)

**Figure 8:**
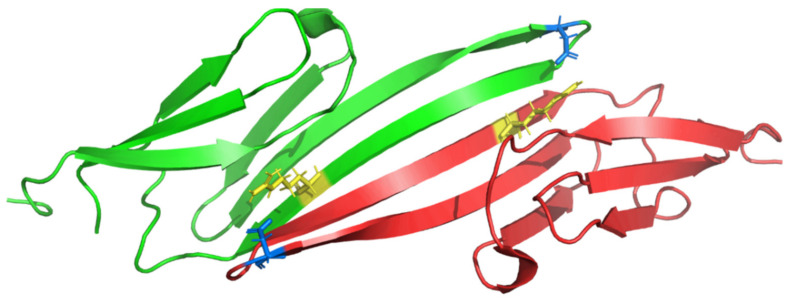
Molecular structure of the αB-crystallin domain determined by nuclear magnetic resonance (NMR) spectroscopy (https://www.rcsb.org/structure/2KLR) (accessed on 13 March 2022) [206]. Two ion bridges are formed between aspartate p.D109 (blue) and arginine p.R120 (yellow) mediating its dimerization. Of note, the mutation CRYAB-p.D109G is associated with RCM in combination with skeletal myopathy [40] and -p.R120G causes MFM in combination with HCM and cataract [19,207].

CRYAB (or HSPB5) encodes αB-crystallin, which belongs to the small heat shock protein (sHSP) family [196]. Several sHSPs are expressed in the human heart. Originally, αB-crystallin was discovered as a major component of the vertebrate eye lenses [197]. However, it is also highly expressed in the heart and in the skeletal muscle [198,199]. In 1998, Vicart et al. identified in a French family with MFM in combination with HCM and cataract the pathogenic missense mutation CRYAB-p.R120G (Figure 8). Of note, this mutation causes, comparable to DES mutation, an abnormal aggregation of desmin and αB-crystallin in skeletal and cardiac myocytes [19]. Interestingly, Sacconi et al. described the same triad of clinical symptoms in a family carrying a different CRYAB mutation [200]. CRYAB mutations can also cause isolated cataract without cardiac involvement [201] or vice versa isolated DCM without cataract [202]. Recently, the CRYAB mutation p.D109G has been described in a small German family with RCM in combination with SM [40]. Interestingly, R120 and D109 form two ion bridges stabilizing the dimerization of αB-crystallin (Figure 8). The αB-crystallin dimers form large oligomers [203] which have an ATP-independent chaperone-like activity [204]. In addition, αB-crystallin binds also to different cytoskeletal and sarcomere proteins, e.g., titin [205].

#### 3.2.8. Bcl2 Associated Athanogene 3 (BAG3) 

The BAG3 gene consists of four exons and encodes Bcl2 associated athanogene 3 [208]. BAG3 is a co-chaperone binding to the ATPase domain of heat shock protein Hsc70/Hsp70 and regulating its chaperone function [209]. BAG3 is structurally organized in an N-terminal tryptophan-tryptophan (WW) domain, two IPV domains, two 14-3-3 binding motifs, a proline-rich region and a C-terminal BAG domain [210,211]. The protein–protein interaction of BAG3 with Hsc70/Hsp70 is mediated by its BAG domain [212]. BAG3 acts as an ATP exchange factor stabilizing the ATPase domain of Hsc70/Hsp70 without bound ATP [213]. Since the multi-domain organization of BAG3, numerous other binding partners have been described. For example, BAG3 binds to several members of the sHSP family including αB-Crystallin [214,215,216,217,218]. Briefly summarized, BAG3 has a central and important role in protein quality control and chaperone-assisted selective autophagy [219].

Several pathogenic mutations in BAG3 have been described in patients with DCM [220] or with MFM [221]. In addition, BAG3 mutations are found in patients with RCM in combination with MFM [42]. Recently, Kimura et al. generated a transgenic mouse model with an overexpression of BAG3-p.P209L conjugated with green fluorescent protein. These mice develop RCM and severe cardiac fibrosis. At the cellular level, disorganization of the Z-disc and abnormal protein aggregation were present [222]. In contrast, the knock-in mouse model carrying the equivalent murine mutation Bag3-p.P215L does not develop a cardiac phenotype [223]. 

#### 3.2.9. Discoidin Cub and Lccl Domain Containing Protein-2 (DCBLD2)

Recently, Alhamoudi et al. described the homozygous nonsense mutation DCBLD2-p.W27X in a 5-year-old Arabic patient with severe RCM, tachycardia, atrial fibrillation, dysmorphic features and developmental delay. Functional analyses using primary dermal fibroblast from the mutation carrier indicated reduced cell proliferation and altered amounts of calcium and reactive oxygen species in comparison to normal fibroblasts [43]. DCBLD2 encodes a ubiquitously expressed type-I transmembrane protein [224,225]. It is involved in vascular smooth muscle cell proliferation [226], vascular endothelial growth factor (VEGF) signaling [227] and epithelial–mesenchymal transition [228]. However, the exact molecular functions of DCBLD2 contributing to RCM and other cardiomyopathies are currently unknown and deserve increased research attention in the future.

## 4. Summary and Outlook

Currently, mutations in over 19 different disease-causing genes have been discovered in patients with primary RCM. However, the genetic landscape of RCM is overlapping with the genetic background of other cardiomyopathies. Genes encoding for sarcomere proteins such as cardiac troponin-I are the major RCM genes. However, more recently, the prevalence of mutations in specific non-sarcomeric genes such as *DES* or *FLNC* has increased; broad NGS gene panels or whole exome sequencing should be considered if a genetic etiology is suspected. This might be also beneficial, since the genetic landscape of RCM remains incomplete. Therefore, multi-center studies enrolling larger patient cohorts are needed to provide a robust overview about the genetic etiology of RCM. In addition, these studies might reveal the age of onset associated with specific genotypes.

As no sufficient treatment for RCM is currently available, there is a highly unmet medical need for the development of more precise genetic or molecular therapies. However, there is hope on the horizon with novel therapies targeting the sarcomere. In particular, for the obstructive form of HCM the allosteric inhibitor of the cardiac specific myosin adenosine triphosphatase (MYK-461) has shown symptomatic improvement in a phase 3 trial and may also be applicable for patients with RCM and sarcomeric mutations leading to an excessive cross bridging with actin [229]. The opposite setting, small molecules, such as omecamtiv mecarbil and danicamtiv, increasing contractility may be effective in particular in patients with sarcomere mutations and DCM [230].

Another exciting strategy can be seen in genome editing using CRISPR-Cas9 [231] or RNA editing using Cas7-11 [232] in combination with adequate cardiomyocyte specific delivery vectors, e.g., adeno-associated viruses [233,234], will help to reach this goal in the future. Recently, CRISPR-Cas9 has been used for example for correcting DCM associated truncating *TTN* mutations [235] and deserves interest in the context of RCM in the future.

## Figures and Tables

**Table 5:** Overview about the known RCM-associated *FLNC* mutations.

Mutation	Clinical Features	Family History	MAF ^1^	Comments	References
p.A1183L	RCM and congenital myopathy	one patient	-		[176]
p.A1186V	RCM and congenital myopathy	three unrelated index patients	-	de novo	[176]
	RCM	one patient	-	de novo, early onset	[182]
p.S1624L	RCM	four affected family members	0.00003		[37]
p.C1639Y	RCM	one patient	-	de novo, early onset	[182]
p.G2011R	RCM	one patient	-	iPSC model	[180]
p.G2151S	RCM	two patients	-	in addition *PTPN11*-p.Q510R	[183]
p.I2160F	RCM	three affected family members	-		[37]
p.V2264M	RCM, SM	one patient	-	iPSC model	[179]
p.V2297M	RCM, AF	five affected family members	0.000004		[181]
p.P2298L	RCM	eight patients (four genotyped)	-		[184]
p.P2301L	RCM, AF, muscular weakness	one patient	-	de novo	[183]
p.Y2563C	RCM	two monozygotic twins	-	de novo	[184]

^1^ MAF = Minor allele frequency according to Genome Aggregation Database (January 2022), https://gnomad.broadinstitute.org/(accessed on 13 March 2022). AF = atrial fibrillation, RCM = restrictive cardiomyopathy, SM = skeletal myopathy.
